# The Relationship Between Neutrophil/Lymphocyte Ratio Before Coronary Angiography and Coronary Collateral Development

**DOI:** 10.7759/cureus.78739

**Published:** 2025-02-08

**Authors:** Sefa Sural, Tarkan Tekten, Hasan Gungor

**Affiliations:** 1 Cardiology, Istinye University, Istanbul, TUR; 2 Cardiology, Adnan Menderes University, Aydın, TUR

**Keywords:** coronary angiography, coronary collateral, lymphocyte, neutrophil, neutrophil/lymphocyte ratio

## Abstract

Objective: The aim of this study was to determine the relationship between the neutrophil/lymphocyte ratio (NLR) measured before coronary angiography and coronary collateral (CC) development.

Method: This retrospective descriptive study was carried out between January 2012 and June 2013 in the cardiology outpatient clinic of a university hospital in Aydın, with 165 patients who were diagnosed with acute coronary syndrome or stable angina pectoris and who had 95% or more stenosis in at least one coronary artery according to angiography. Coronary artery stenosis was determined by Gensini scoring. The classification of CC was performed by the Rentrop method, and patients were divided into two groups: Rentrop stages 0 and 1 (poor CC filling (group 1)) and Rentrop stages 2 and 3 (good CC filling (group 2)). The data were analyzed using appropriate statistical analyses. Multivariate logistic regression was used to determine the predictors of CC level, and receiver operating characteristic (ROC) curve analysis was performed to calculate the predictive value of predictors.

Results: When the groups were compared, the mean age (p=0.023), Gensini score (p<0.001), smoking status (p=0.012), creatinine level (p=0.032), and aspartate aminotransferase level (p=0.032) of the patients in group 1 were significantly lower than those in group 2, while their total cholesterol (p=0.006) and low-density lipoprotein (LDL) levels (p=0.020) were higher. Neutrophils (p=0.016) and NLR (p<0.001) were significantly higher in group 2 patients. A significant positive correlation was found between CC level and neutrophils (p=0.035) and NLR (p=0.011). In regression analysis, high NLR, high Gensini score, and smoking were predictors of good CC filling. According to ROC curve analysis, the sensitivity and specificity of NLR ≥3.53 at the time of presentation to the clinic for predicting good CC filling were 37.6% and 85%, respectively.

Conclusion: This study showed that NLR was significantly associated with the development of good CC filling; as NLR increased, the development of good CC filling increased in patients.

## Introduction

Cardiovascular disease (CVD) is the leading cause of death both worldwide [1] and among the known causes of death [2]. While a total of 1,055,000 cases, including 720,000 new and 335,000 recurrent cases, were detected in the United States in 2019, CVD is the most common cause of death at 35.4%, according to the 2022 data from the Turkish Statistical Institute (TUIK). CVD, having high morbidity and mortality rates, is an important public health problem. Risk determination and early diagnosis are essential, and treatment must be started as soon as possible [2-4].

Coronary artery disease (CAD), which accounts for more than 90% of CVD [5], is characterized by the inability to provide blood flow, which is essential for the metabolic needs of the heart muscle, as a result of the occlusion of the coronary arteries by atheromatous plaques [6].

Coronary collaterals (CC) appear as intervascular anastomotic connections that develop as an adaptive structure to ensure blood flow to ischemic areas in the heart due to atherosclerosis. These structures cannot be visualized angiographically until the stenosis in the coronary arteries exceeds 90%. In case of complete occlusion of the coronary artery, it supplies 50% of the flow and helps meet the increased oxygen demand of the myocardium, thus creating protected areas [7]. Thus, by limiting myocardial ischemia and infarction areas, it allows the viability of the myocardium to continue for a long time [8,9].

The impact of atherosclerosis and the inflammation process on cardiovascular events remains unclear [10]. Cells in the atheroma plaque accumulated in the artery wall due to atherosclerosis; it constitutes the source of pro-inflammatory cells such as chemokines, cytokines, and platelet-activating factors. It is known that these cells stimulate inflammation within the plaque, leading to the progression of lesions [11]. In the literature, it is stated that the inflammation process is effective in the development of atherosclerosis [12,13]. Good CC development is observed in patients with high serum monocyte levels [14]. Contradictory results have been reported, such as an inverse relationship between the high-sensitivity C-reactive protein (hs-CRP) rate and the level of CC development [15]. Another study has shown that white blood cell (WBC) levels and subtypes (neutrophils, monocytes, and lymphocytes) are predictors of CAD. Leukocytes, especially neutrophils, play an important role in atherogenesis and atherothrombosis. The neutrophil/lymphocyte ratio (NLR) has been suggested as a prognostic marker to determine the systemic inflammatory response [16]. Based on the contradictory results in the literature, this research aimed to determine the relationship between NLR, a marker of inflammation, before coronary angiography and the level of CC development. This ratio can be used to predict morbidity, mortality, and myocardial infarction in high-risk CAD and to improve its management.

## Materials and methods

**Figure 1:**
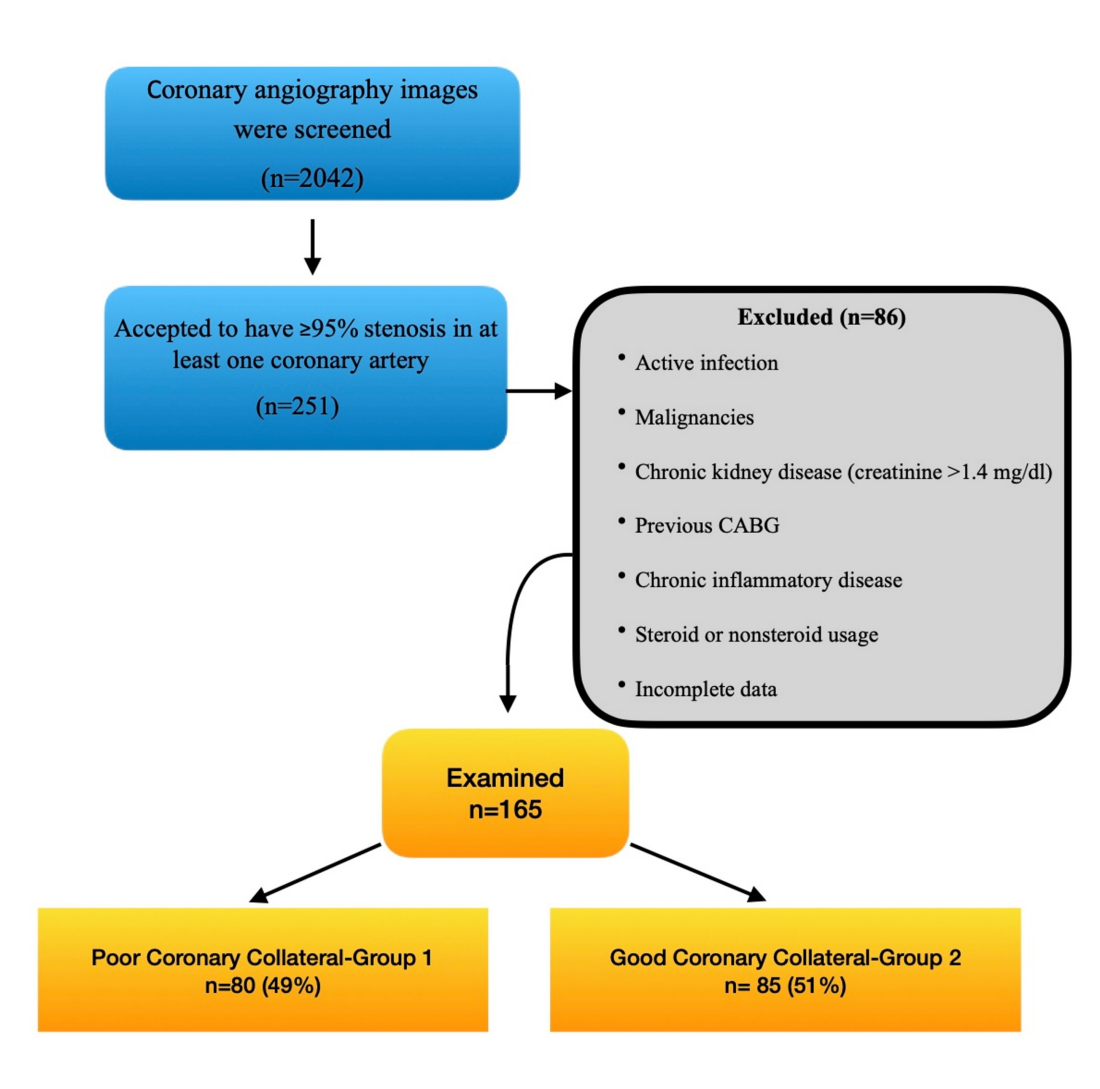
Study flow diagram CABG: coronary artery bypass grafting

In this retrospective study, clinical records and echocardiography, angiography, and laboratory findings in the files of 165 patients diagnosed with CAD in the Cardiology Department of Adnan Menderes University in Aydın between January 2012 and June 2013 were examined as shown in Figure 1. Patients diagnosed with CAD (acute coronary syndrome or stable angina pectoris) and with ≥95% stenosis in at least one coronary artery as a result of coronary angiography were included in the study. Acute coronary syndromes were accepted as patients diagnosed with unstable angina, ST-segment elevation myocardial infarction (STEMI), and non-STEMI. The condition of angina not observed at rest occurring with the same character for weeks without significant changes in frequency, severity, and duration was defined as stable angina (no recurrence of chest pain at rest).

Patients' age, gender, hypertension (blood pressure >140/90 mmHg), diabetes (fasting blood sugar ≥126 mg/dl or at least one measurement value ≥200 mg/dl), hyperlipidemia (history of using any lipid-lowering medication), and characteristic findings such as smoking history, alcohol use history, and family history (first-degree male relatives <45 years of age or first-degree female relatives <55 years of age diagnosed with CAD) were evaluated. In addition, the medications used before angiography and previous treatment attempts (percutaneous coronary intervention, coronary artery bypass grafting (CABG), medical treatment) were recorded.

Angiographic procedures were performed in a single center, in the same intervention unit, and with the same brand of device. Coronary arteries were visualized using cranial and caudal angles in the right and left oblique planes. Coronary angiographies and collateral grading were reviewed by three experienced interventional cardiologists who were unaware of the clinical characteristics and laboratory results of the patients. In case of failure to reach a consensus, disagreements between the three cardiologists were resolved based on the opinions of two of the three (2/3) cardiologists. The degree of stenosis of the coronary arteries was decided jointly with the approval of three physicians, based on the angle at which the stenosis was most visible.

Patients with active infection, a diagnosis or history of malignancy, a previous history of CABG, a diagnosis of renal failure, active or previous use of nonsteroidal or steroid drugs, and a history of chronic rheumatological disease and patients who have incomplete data were not included in the study. Active infection was defined as a fever over 38°C with or without a focus of infection or use of antibiotics due to a diagnosed infection.

Stenosis in coronary arteries was graded using the Gensini score, a more meaningful scoring system for determining the severity of coronary heart disease [17]. Using this scoring system, 1 point was given for 1-25% narrowing, 2 points were given for 26-50% narrowing, 4 points were given for 51-75% narrowing, 8 points were given for 76-90% narrowing, 16 points were given for 91-99% narrowing, and 32 points were given for 100% blockage. Subsequently, each score has been multiplied by the coefficients determined according to the region of the lesion (left main coronary artery (LMCA) lesion ×5, left anterior descending (LAD) and circumflex (CFX) proximal lesion ×2.5, LAD middle region ×1.5, LAD distal, first diagonal, right coronary artery (RCA) proximal, middle, and distal, first optus marginalis, and CFX middle lesions ×1, and second diagonal and posterolateral branch ×0.5) and then added to determine the final Gensini score [17].

The Rentrop method, which is the most appropriate angiographic method for evaluating CC circulation, was used [18]. It is comprised of several stages. In Rentrop stage 0, there is no CC filling. In Rentrop stage 1, there is barely detectable CC filling. The contrast material partially passes into the collateral vessels. However, epicardial vessels cannot be visualized. In Rentrop stage 2, there is partial CC filling. The contrast material passes into the collateral vessels, but epicardial coronary arteries cannot be fully visualized. In Rentrop stage 3, there is complete perfusion in the coronary artery. The contrast material passes into the collateral vessels, and the epicardial coronary artery becomes fully visible [19]. Patients were divided into two groups: poor CC filling as group 1 (Rentrop stages 0 and 1) and good CC filling as group 2 (Rentrop stages 2 and 3).

Written permission was obtained from the Adnan Menderes University Ethics Committee (approval number: 2013/244; date: 13.06.2013) to conduct the research. Informed consent was not obtained due to the study's retrospective design. The research was carried out within the framework of the principles of the Declaration of Helsinki.

Statistical analysis

All data were analyzed in a computer environment using SPSS Statistics for Windows, Version 17.0 (Released 2008; SPSS Inc., Chicago, Illinois, United States). Continuous variables were expressed as mean±standard deviation and categorical variables as percentage (%). The t-test was used to compare parametric variables, and the Mann-Whitney U test was used to compare nonparametric values. The Kolmogorov-Smirnov test was employed to assess the normality of the distribution of continuous variables. The Pearson correlation analysis was performed to examine the correlation between CC level and hematological parameters. Multivariate logistic regression analysis was performed to determine the predictors of CC level. Receiver operating characteristic (ROC) curve analysis is an analysis method that will contribute significantly to the clinical decision-making process by determining appropriate cut-off points for markers that can be easily obtained in a short time and at a low cost in cases where the diagnostic process is long and costly and requires special methods, equipment, and qualified manpower. The ability of the test to distinguish true positives is defined as sensitivity, while the ability to distinguish true negatives is defined as specificity. Grading is used in the interpretation of these values ​​(1.00-0.90: excellent; 0.90-0.80: good; 0.80-0.70: medium; 0.70-0.60: poor; 0.60 and below: unsuccessful). The ROC curve analysis results obtained from this study show that the specificity of the NLR value in predicting the level of CC filling is unsuccessful (37.6%), but it has a good level of sensitivity (85%). In the study, a p-value of <0.05 was considered statistically significant.

## Results

A total of 165 patients were included in this study. The average age of the patients was 61.18±10.57 years and 127 (77%) patients were male. There was good CC circulation in 85 (51%) of the patients (group 2); it was determined that 80 (48.5%) patients developed poor CC circulation (group 1).

Table 1 presents a comparison of the clinical, demographic, and laboratory data for patients categorized by their CC, specifically between those with poor and good CC. Notably, patients with poor CC were younger than their counterparts in the good CC group (p=0.023), and there was a higher prevalence of male patients in the poor CC category (67.5% vs. 85.9%; p=0.006). The prevalence of hypertension, hyperlipidemia, diabetes mellitus, a family history of CAD, alcohol use, peripheral arterial disease, and chronic obstructive pulmonary disease was found to be similar across both groups. Interestingly, smoking rates were higher among patients in the good CC group (68.2% vs. 48.8%; p=0.012). The Gensini score was also significantly elevated in the good CC group (p<0.001).

**Table 1:** Patient characteristics and clinical data ^α^Pearson chi-squared test; ^β^t-test Values are in the form of mean±SD or n (%). SD: standard deviation; CC: coronary collateral; CAD: coronary artery disease; COPD: chronic obstructive pulmonary disease; PAD: peripheral arterial disease; M: male; F: female

	Group 1 (poor CC), mean±SD (n=80)	Group 2 (good CC), mean±SD (n=85)	Test value	P-value
Age (years)	61.06±9.33	61.28±11.65	5.283^β^	0.023
Gensini score	35.53±18.54	52.83±24.70	0.000	<0.001
Male/female, (n)	54/26	73/12	7.856^α^	0.006
Medical history				
Diabetes mellitus, n (%)	23 (28.7%)	30 (35.3%)	0.809^α^	0.407
Hypertension, n (%)	39 (48.8%)	47 (55.3%)	0.707^α^	0.438
Hypercholesterolemia, n (%)	16 (45.7%)	19 (22.4%)	0.137^α^	0.849
Smoker, n (%)	39 (48.8%)	58 (68.2%)	6.458^α^	0.012
Family history of CAD, n (%)	6 (7.5%)	4 (4.7%)	0.565^α^	0.526
PAD, n (%)	0 (0%)	4 (4.7%)	3.858^α^	0.121
Alcohol use, n (%)	1 (1.2%)	2 (2.4%)	0.281^α^	1.000
COPD, n (%)	4 (5%)	8 (9.4%)	1.189^α^	0.372

The usage of medications such as angiotensin-converting enzyme inhibitors, angiotensin receptor antagonists, calcium channel blockers, clopidogrel, statins, fibrates, nitrates, diuretics, and acetylsalicylic acid was comparable between the two groups (Table 2).

**Table 2:** Drug usage of the study groups ^α^Pearson chi-squared test CC: coronary collateral; ACE: angiotensin-converting enzyme

Drug usage	Group 1 (poor CC), n (%)	Group 2 (good CC), n (%)	Test value	P-value
ACE inhibitors	19 (23.8%)	30 (35.3%)	0.211^α^	0.126
Beta-blockers	30 (40%)	37 (43.5%)	0.752^α^	0.752
Angiotensin receptor antagonists	10 (12.5%)	11 (12.9%)	0.007^α^	1.000
Calcium channel blocker	8 (10%)	14 (16.5%)	1.493^α^	0.257
Clopidogrel	15 (18.8%)	21 (24.7%)	0.857^α^	0.451
Statin	25 (31.2%)	37 (43.5%)	2.649^α^	0.111
Nitrate	7 (8.8%)	8 (9.4%)	0.022^α^	1.000
Furosemide	0 (0%)	3 (3.5%)	2.876^α^	0.246
Acetylsalicylic acid	31 (38.8%)	42 (49.4%)	1.899^α^	0.210
Fibrate	0 (0%)	1 (1.2%)	0.947^α^	1.000
Spironolactone	0 (0%)	1 (0%)	0.947^α^	1.000

Upon analyzing the echocardiographic parameters, it was noted that measurements of left ventricular end-systolic diameter (LVESd), left ventricular end-diastolic diameter (LVEDd), interventricular septum (IVS), left ventricular (LV) posterior wall, and aortic root were significantly greater in the group with good CC (all p<0.05). In terms of biochemical laboratory parameters, serum creatinine and aspartate aminotransferase levels were elevated in the good CC group, whereas total cholesterol and low-density lipoprotein (LDL)-cholesterol levels were higher in the poor CC group. Complete blood count analysis revealed that the neutrophil count (6.52±2.77 vs. 5.48±1.87×10^3^/mm^3^; p=0.016) and the NLR (3.22±1.62 vs. 2.44±1.02; p<0.001) were significantly greater in the good CC group. The two groups were comparable with regard to other laboratory values. Additionally, a negative correlation was observed between hemoglobin levels and CC circulation, whereas increased neutrophil count (p=0.035) and the NLR (p=0.011) demonstrated a significant positive correlation with good CC (Table 3 and Table 4).

**Table 3:** Laboratory parameters, hematological parameters, and echocardiographic findings GFR: glomerular filtration rate; AST: aspartate aminotransferase; ALT: alanine aminotransferase; LDL-C: low-density lipoprotein-cholesterol; HDL-C: high-density lipoprotein cholesterol; WBC: white blood cell; RDW: red blood cell distribution width; PLT: platelet; MPV: mean platelet volume; NLR: neutrophil/lymphocyte ratio; EF: ejection fraction; LVESd: left ventricular end-systolic diameter; LVEDd: left ventricular end-diastolic diameter; IVS: interventricular septum; SPAP: systolic pulmonary arterial pressure; TR: tricuspid regurgitation; MR: mitral regurgitation; AR: aortic regurgitation; NA: not applicable

	Group 1 (poor CC), mean±SD	Group 2 (good CC), mean±SD	Reference range	P-value
Laboratory parameters				
Glucose, mg/dl	138.17±66.84	157.12±109.50	70-109	0.349
Urea, mg/dl	34.30±12.41	36.81±15.13	16.6-48.5	0.249
Creatinine, mg/dl	0.83±0.16	0.93±0.32	0.6-1.2	0.032
Sodium, meq/l	138.18±3.13	137.24±2.88	135-150	0.050
GFR, ml/min	95.32±21.20	92.84±20.66	90-120	0.449
Potassium, meq/l	4.21±0.42	4.32±0.49	3.5-5.5	0.103
Magnesium, meq/l	2.08±0.20	2.11±0.29	1.7-2.2	0.537
Calcium, meq/l	9.28±0.62	9.07±0.54	8.6-10.3	0.051
Total bilirubin, mg/dl	0.55±0.25	0.59±0.29	0.3-1.2	0.524
Direct bilirubin, mg/dl	0.20±0.08	0.22±0.11	0.1-0.3	0.751
AST, U/L	34.03±36.00	59.83±72.82	8-37	0.032
ALT, U/L	25.14±13.08	29.85±33.02	4-36	0.445
Total cholesterol, mg/dl	196.98±39.86	178.74±35.51	50-200	0.006
LDL-C, mg/dl	123.79±36.20	110.47±28.23	60-130	0.020
HDL-C, mg/dl	37.86±11.21	37.59±11.49	>35	0.889
Triglyceride, mg/dl	179.72±108.02	157.93±95.65	40-150	0.101
Hematological parameters				
Hemoglobin, g/L	13.87±1.59	13.58±1.53	13.5-17.5	0.239
Hematocrit, %	41.17±6.36	41.15±4.23	40.1-52.0	0.978
WBC count, ×10^3^/mm^3^	8.86±2.45	9.53±3.99	4.0-10.0	0.482
RDW, %	14.65±2.22	14.69±1.71	11.8-16.1	0.468
PLT, ×10^3^/mm^3^	268.28±101.61	263.28±745.74	150-450	0.206
MPV, fl	8.83±0.87	8.65±1.11	6.8-10.8	0.221
Neutrophil, ×10^3^/mm^3^	5.48±1.87	6.52±2.77	3.4-6.8	0.016
Lymphocyte, ×10^3^/mm^3^	2.50±1.02	2.24±0.88	2.1-5.4	0.112
NLR	2.44±1.02	3.22±1.62	NA	<0.001
Echocardiographic findings				
EF, %	52.5±10.6	50.1±11.0	50-70	0.127
Left atrium, mm	36.2±4.5	37.5±5.7	25-40.8	0.232
LVESd, mm	32.6±5.6	35.5±6.1	20.4-37	0.021
LVEDd, mm	49.0±4.8	51.2±4.6	35.2-56.1	0.035
IVS, mm	10.9±1.6	11.4±1.5	6.1-11.8	0.027
Posterior wall, mm	10.5±1.2	10.9±1.3	6.0-10.0	0.017
Aortic root, mm	30.1±3.6	32.2±3.3	19.8-33.5	0.008
SPAP, mmHg	34.4±10.5	32.6±8.2	15-30	0.523
TR, level	1.1±0.4	1.2±0.5	NA	0.133
MR, level	1.4±0.5	1.6±0.7	NA	0.138
AR, level	1.2±0.4	1.3±0.4	NA	0.948

**Table 4:** Relationship between hematological parameters and development level of CC circulation MPV: mean platelet volume; RDW: red blood cell distribution width; WBC: white blood cell; NLR: neutrophil/lymphocyte ratio; CC: coronary collateral

Hematological parameters	Correlation coefficient (r)	P-value
Hemoglobin	-0.158	0.043
Hematocrit	-0.045	0.570
Platelet	0.000	0.998
MPV	-0.115	0.143
RDW	0.039	0.321
WBC count	0.088	0.262
Neutrophil	0.165	0.035
Lymphocyte	-0.078	0.321
NLR	0.197	0.011

In multivariate logistic regression analysis, increased NLR (p=0.003) and Gensini score (p<0.001) were identified as independent predictors of good CC after adjusting for potential confounders (Table 5).

**Table 5:** Predictors of CC circulation *Logistic regression analysis shows P-value NLR: neutrophil/lymphocyte ratio; CC: coronary collateral

Variables	Odds ratio	Confidence interval (95%)	P-value*
NLR	0.083	0.029-0.136	0.003
Gensini score	0.007	0.003-0.011	<0.001
Smoker	0.166	0.010-0.323	0.038

**Figure 2:**
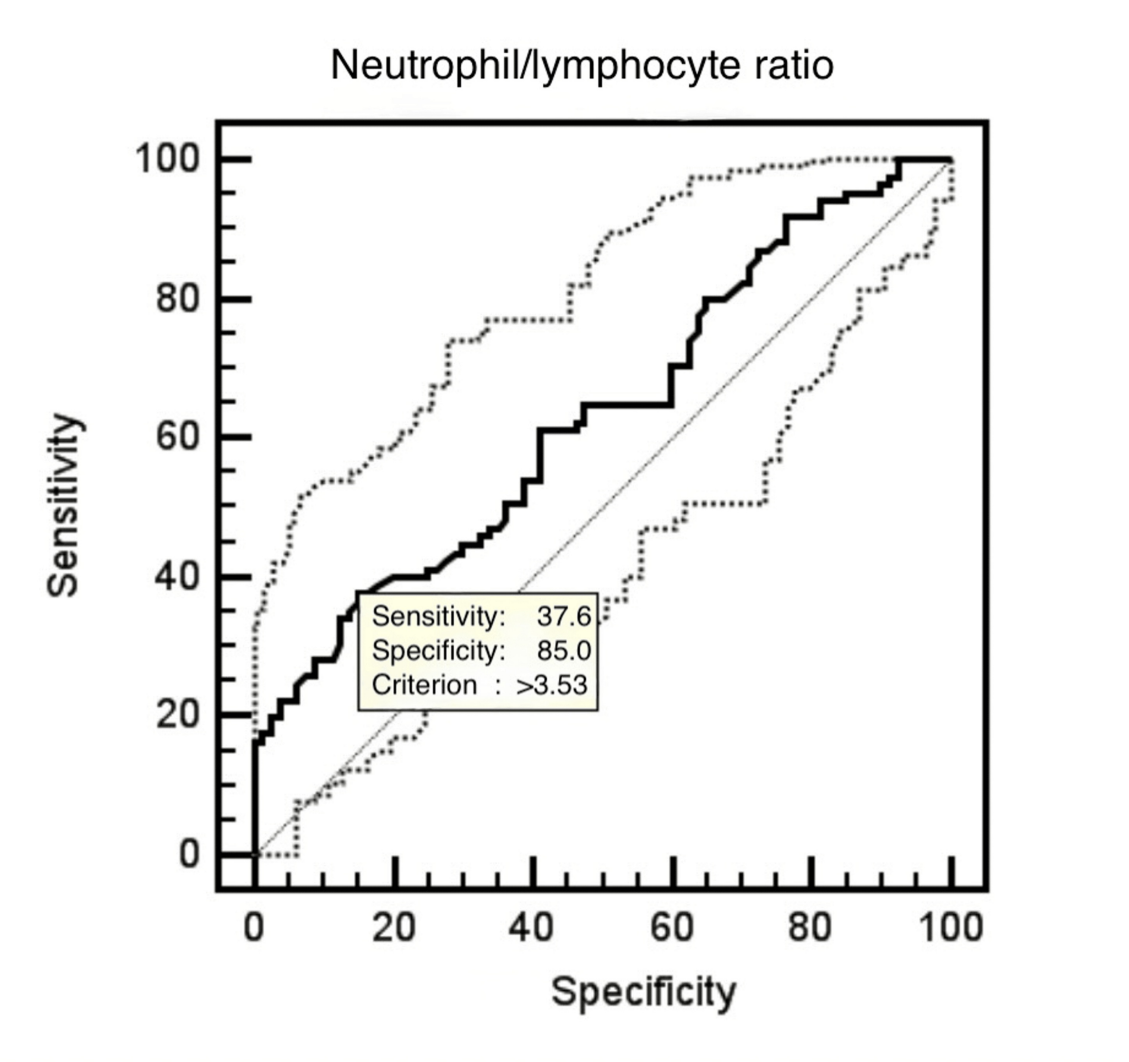
ROC curve analysis ROC: receiver operating characteristic

The ROC curve analysis demonstrated that an NLR threshold of 3.53 predicted good CC with a sensitivity of 37.6% and a specificity of 85% with the area under the ROC curve being 0.632 (95% CI: 0.55-0.71, Figure 2).

## Discussion

Although CAD is more common in men than in women, it becomes more common in all individuals with advancing age due to the loss of elasticity in the vessels [20].

There are many studies in the literature showing a significant relationship between the development of CC and CAD risk factors such as age, diabetes, hyperlipidemia, hypertension, and smoking [21,22]. In this study, no significant relationship was found between diabetes, hyperlipidemia, hypertension, and CC filling level among the groups, but similar to the literature, male gender, advanced age, and smoking were found to have a significant relationship with CC filling level. This result may have caused more coronary artery stenosis and better coronary artery filling levels in men, smokers, and elderly patients. It is thought that many mechanical and biochemical factors such as recurrent ischemia, increased shear stress, pressure gradient, and angiogenic growth factors may be associated with the development of collateral arteries [19]. During arterial occlusion, growth factors that are regulated under strict control are activated [23]. Growth factors, proteases, protease inhibitors, and cytokines released from activated macrophages and endothelial cells play an important role in the formation of collaterals. Vascular endothelial growth factor (VEGF) and fibroblast growth factor (FGF) are the most emphasized growth factors in the formation of CC circulation. VEGF is released by vascular smooth muscle cells, myocardial cells, and monocytes in response to hypoxia. It is known that smoking accelerates the atherosclerotic process, triggers inflammation, and increases ischemia [22]. The finding of a significant relationship between smoking and CC development in our study supports this result.

The mean platelet volume (MPV) value is an indicator of the activity of platelets; platelets are involved in plaque rupture and thrombus development during myocardial infarction. There are conflicting results in the literature regarding the relationship between CC circulation development and MPV value [24-26]. No relationship was found between MPV value and CC development in this study. High cholesterol levels negatively affect the development of CC by slowing down angiogenesis [21]. In this study, total cholesterol and LDL levels in patients with good CC circulation were significantly lower than in patients with poor CC circulation. This result suggested that angiogenesis was not affected in the group with good CC circulation. A key finding of our study was the negative correlation between hemoglobin levels and the presence of good CC. Previous research, grounded in anatomical observations, has shown that anemia can enhance intracoronary collateralization [27]. It has been suggested that this occurs due to increased blood flow resulting from reduced blood viscosity and heightened tissue hypoxia; however, the precise underlying mechanism remains poorly understood. Additionally, prior clinical studies have indicated a connection between anemia and improved CC, although the physiological mechanisms have not been definitively established [28]. Our study's findings further corroborate this evidence.

The relationship between the Gensini score and CC circulation has not been extensively studied. However, the limited available literature suggests a positive correlation between a higher Gensini score and good CC [29]. Our study's findings align with these previous reports, further supporting this association.

Although neutrophil count, lymphocyte count, and NLR are markers of systemic inflammation [30,31], C-reactive protein (CRP) is also accepted as a marker of the vascular inflammation process [32,33]. During the inflammatory response, the number of neutrophils increases and is accompanied by relative lymphopenia [30,31]. There are studies with conflicting results showing the relationship between CC circulation development and inflammation. Increasing the number of neutrophils increases the proliferation and migration of endothelial cells [34,35]. High CRP level negatively affects CC development as it prevents vascular endothelial cell migration [15,36,37]. It has been reported that high monocyte levels [38] and serum adiponectin levels positively affect the development of CC [14]. However, the relationship between NLR and CC development is not clear. A study [16] in the literature highlights a significant relationship between NLR and poor CC, suggesting that the NLR may serve as a predictor for the development of inadequate CC circulation. However, other studies have reported no relationship between NLR and CC circulation development [39]. Çil et al. [39] explored the association between NLR and CC circulation in a cohort of 82 patients undergoing angiography. This descriptive study concluded no relationship was found between NLR and the progression to good or poor CC circulation, citing the limited sample size as a significant constraint. Conversely, Ayhan et al. [24] conducted a retrospective analysis involving 96 patients and identified that an elevated NLR was predictive of poor CC circulation development. In a study by Uysal et al. [16], which included 521 patients (226 with good CC circulation and 295 with poor), a link was established between NLR and the development of poor CC circulation. This study suggested that the NLR could predict poor CC circulation development with a sensitivity of 65% and specificity of 68%. In this research, contrary to the literature, a significant correlation was found between NLR level and good CC circulation development. Moreover, according to ROC curve analysis, the 85% specificity of the NLR value measured at the time of admission to predict the CC level supports this result. Finding this significance suggested that the fact that lymphocyte values ​​remained constant while neutrophil values ​​were significantly higher in patients with good CC circulation may have been effective. However, the fact that NLR, one of the inflammation markers, accelerates atherosclerosis and triggers ischemia may have supported the development of good CC circulation. Increased NLR is a well-established marker of systemic inflammation. The development of good CC circulation is known to be promoted by chronic ischemia. Previous studies have reported elevated inflammatory markers in patients with chronic myocardial ischemia, supporting the link between sustained ischemia and inflammatory activation [40]. Although some studies have presented conflicting results, our findings align with the notion that chronic myocardial ischemia is associated with increased inflammation and, consequently, higher NLR levels, which may contribute to the development of good CC. Given that NLR is a readily accessible and cost-effective parameter available in any healthcare setting, its evaluation may provide valuable preliminary insights into identifying patients likely to exhibit good CC.

CC circulation reduces ischemia and limits the ischemic area by providing perfusion distal to the lesion causing ischemia [41,42]. Therefore, CC circulation positively affects LV functions and increases survival rates [43]. In the literature, it has been reported that CC circulation has positive effects on LV systolic functions, that the prevalence of CAD is high [44], and that LV ejection fraction (LVEF) is lower in the group with good CC circulation [45]. In this study, contrary to the literature, there was no statistically significant difference in systolic functions between the groups, but LVESd and LVEDd were higher in the group with good CC circulation. It is well established that increased LV diameter may indicate LV hypokinesia. Previous studies have reported that LV wall motion abnormalities are commonly observed in patients with chronic coronary ischemia and chronic ischemia itself is recognized as a stimulus for developing good CC [46]. In this context, our findings indirectly support these prior observations. Furthermore, observational studies have demonstrated an association between increased LV mass index (LVMI) and good CC in patients with chronic total occlusion [47]. In our study, the higher values of IVS and LV posterior wall thickness and increased LVESd and LVEDd in the group with good CC suggest an elevated LVMI in this cohort. These findings are consistent with previous analyses, further reinforcing the relationship between LV structural remodeling and the presence of good CC.

Previous studies have demonstrated that anti-ischemic drug therapies positively influence CC circulation [48-50]. However, in our patient cohort, no significant correlation was observed between anti-ischemic drug use and good CC. This lack of association may be attributed to the relatively small sample size and the balanced distribution of drug use between the study groups.

Study limitation

This research was conducted in a single institution and the results are limited to the sample at the institution. Aside from its retrospective design which is the main limitation of our study, it also has several other limitations. The data are based on files that were not designed to collect data for the study, so it is inevitable that data cannot be comprehensively collected. Patient selection and recall biases may also affect the results, and the reasons for treatment differences between patients are often not identified and may lead to bias. The other important limitation is the small number of patients since our study was performed within a limited period of time (January 2012-June 2013). Additionally, standard measurements may not have been obtained due to echocardiographic evaluations performed by different cardiologists and methodological errors.

## Conclusions

In this study, it was determined that age, male gender, and smoking, which are among the risk factors of CAD, were effective in the development of CC circulation. Multiple factors contributing to ischemia influence the extent of CC development. These include advanced age, smoking, higher Gensini scores, and anemia. The interplay of these factors likely enhances the ischemic stimulus, thereby promoting the formation of CC circulation. NLR value, one of the inflammation markers, accelerates atherosclerosis and triggers ischemia. It was observed that CC affected the development of circulation. Based on the results obtained, NLR value can be used clinically as a predictor to determine good CC circulation development. Compared to other systemic immunomarkers, the NLR is easily obtainable, widely accessible across healthcare institutions, and cost-effective. As a laboratory parameter that provides insight into systemic inflammation and was independently associated with good CC circulation in our study, NLR may serve as a valuable tool in guiding treatment strategies for patients with ischemia-related symptoms and a diagnosis of chronic coronary syndrome. For our study's findings to be applicable in clinical practice, they must be validated through multicenter, prospective studies with larger patient populations. Such investigations would strengthen the reliability and generalizability of our results, ensuring their broader clinical utility.
